# A Smartphone Integrated Platform for Ratiometric Fluorescent Sensitive and Selective Determination of Dipicolinic Acid

**DOI:** 10.3390/bios12080668

**Published:** 2022-08-22

**Authors:** Xiang Li, Junsong Wu, Huaguang Hu, Fangfang Liu, Jialian Wang

**Affiliations:** 1 School of Marine and Biological Engineering, Yancheng Teachers University (YCTU), Yancheng 224002, China; 2 Department of Basic Medical Science, Jiangsu Vocational College of Medicine, Yancheng 224005, China

**Keywords:** ratiometric, fluorescence, determination of dipicolinic acid, smartphone-based detection

## Abstract

A desirable lanthanide-based ratiometric fluorescence probe was designed as a multifunctional nanoplatform for the determination of dipicolinic acid (DPA), a unique bacterial endospore biomarker, with high selectivity and sensitivity. The carbon dots (CDs) with blue emission wavelengths at 470 nm are developed with europium ion (Eu^3+^) to form Eu^3+^/CDs fluorescent probes. DPA can specifically combine with Eu^3+^ and then transfer energy from DPA to Eu^3+^ sequentially through the antenna effect, resulting in a distinct increase in the red fluorescence emission peak at 615 nm. The fluorescence intensity ratio of Eu^3+^/CDs (fluorescence intensity at 615 nm/fluorescence intensity at 470 nm) showed good linearity and low detection limit. The developed ratiometric nanoplatform possesses great potential for application in complex matrices owing to its specificity for DPA. In addition, the integration of a smartphone with the Color Picker APP installed enabled point-of-care testing (POCT) with quantitative measurement capabilities, confirming the great potential of the as-prepared measurement platform for on-site testing.

## 1. Introduction

Inhalation of 10^4^ or more concentration of *Bacillus anthracis* (*B. anthracis*) spores can lead to a mortality of as high as 75%, even within 24 to 48 h of drug treatment [1,2], indicating that *B. anthracis* spores poses a serious threat to human health and public safety [3,4]. Therefore, sensitive and selective determination of *B. anthracis* spores had a critical role to play in early clinical diagnosis and preventing disease outbreaks [5,6]. Dipicolinic acid (DPA), making up approximately 5–15% of the dry weight of anthrax spores, was an extremely important component and major biomarker of anthrax spores [7,8,9,10]. Consequently, the level of anthrax spores could be detected and evaluated through the concentration of DPA. Recently, although considerable strategies for the determination of DPA had been constructed, including electrochemical assays, Surface-enhanced Raman spectroscopy (SERS), mass spectrometry (MS), colorimetric methods, etc. [11,12]. These strategies are usually cumbersome, time-consuming and require large instruments, which may not be suitable for on-site rapid detection of DPA. In general, fluorescence-based sensing was a promising optical candidate for addressing the above-mentioned problems in DPA determination [13,14,15]. However, the further application of fluorescent probes was greatly restricted by the shortcomings of their used currently, such as complicated synthesis processes, poor fluorescence stability and low sensitivity [16].

Lanthanide-based fluorescence nanoprobes had been constructed and employed to the determination of targeting on account of that special optical properties such as large Stokes shifts, long fluorescent lifetimes and high fluorescence stability [17,18,19,20]. In fluorescence measurements, DPA acted as a single ligand coordinated with Eu^3+^ or terbium ions (Tb^3+^) and then sensitized lanthanide ions emission by transferring excitation energy to the lanthanide ions by an “antenna effect”, a strategy that can be employed to sensing of DPA levels [21,22]. Ratiometric fluorescence detection strategies, recording fluorescence emission at two different wavelengths, had attracted increasing attention and interest from researchers because of the increased accuracy and reproducibility of target assays as well as the reduction of false positive or false negative signal results compared to strategies with a single emission signal [23,24,25,26,27].

**Scheme 1:**
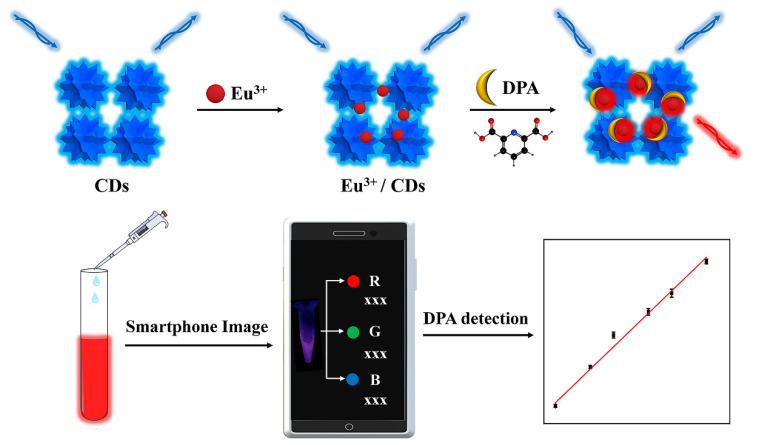
Llustration of the ratiometric fluorescence sensing for DPA detection.

Herein, a desirable ratiometric fluorescence platform was fabricated for the highly sensitive and selective detection of the *B. anthracis* biomarker DPA by integrating Eu^3+^ and CDs (Eu^3+^/CDs) in Scheme 1. Taking into consideration fully the coordination binding of DPA to Eu^3+^ in the complex of Eu^3+^/CDs, the formed complex of Eu^3+^/DPA then emitted a red fluorescence while the blue fluorescence of CDs was not changed. Therefore, the fluorescence ratio (F_615_/F_470_) of red fluorescence to blue fluorescence as a signal can be augmented with increasing concentrations of DPA. Furthermore, the developed strategy was further utilized for the sensitivity detection of DPA in real samples by integrating a smartphone with Color Picker APP. The ratiometric fluorescence sensing platform based on Eu^3+^/CDs fluorescent probes has the ability to rapidly detect DPA in the field and thus assess the infection level of anthrax spores, which has great potential in food safety and healthcare.

## 2. Materials and Methods

### 2.1. Materials and Reagents

Chemicals including Eu(NO_3_)_3_⋅6H_2_O, dipicolinic acid (DPA) were purchased from Aladdin Reagent Co., Ltd. (Shanghai, China). Polyethylenimine was purchased from Sigma-Aldrich. Citric acid, glutamic acid (Glu), cysteine (Cys), glutathione (GSH), adenosine triphosphate (ATP), sodium chloride (KCl), ferric chloride hexahydrate (FeCl_3_·6H_2_O), zinc nitrate (ZnNO_3_) and nickel chloride hexahydrate (NiCl_2_·6H_2_O) were purchased from Sinopharm Group Chemical Reagent Co., Ltd. Milk was purchased from the local supermarket.

### 2.2. Preparation of CDs

CDs were synthesized according to reported work [28]. First, the citric acid and polyethylenimine was dissolved in 10 mL DI water under stirring. Then, the mixture solution was poured into a poly(p-phenol)-lined stainless-steel autoclave and heated at 200 °C for 5 h. After that, the obtained solution was dialyzed to remove unreacted impurities.

### 2.3. Determination of DPA with Eu^3+^/CDs Sensing System

To determine the optimal incubation time, the DPA (100 μM) was incubated and stirred with Eu^3+^/CDs in Phosphate Buffer (PB) (10 mM, pH = 7.5) for different minutes (1–20 min) at 25 °C. Then, the fluorescence intensity of the sensing system was recorded by fluorescence spectrometry at 285 nm excitation. Similarly, only the incubation temperature (25–55 °C) was changed in above sensing system to determine the optimal incubation temperature. After adding Eu^3+^/CDs into PB, the DPA with different concentrations (0–200 μM) was added above the solution and stirred for 10 min at 25 °C. Then, the fluorescence intensity of the sensing system was recorded by fluorescence spectrometry at 285 nm excitation. For interference experiments, the DPA, GSH, Cys, Glu, ATP, Cl^−^, Zn^2+^, Ni^2+^ and Fe^3+^ were added to Eu^3+^/CDs solution, respectively. The final concentration of DPA and other interfering substances was 50 μM. Finally, the emission spectra at 285 nm excitation of these reaction solutions were recorded.

### 2.4. DPA Sensing in Real Samples

As for DPA detection in real samples, milk was selected as samples. The purchased milk was treated in the same way as previously reported [29]. In order to remove proteins, the purchased milk was sonicated for 30 min with trichloroacetic acid and then the mixture was centrifuged for 5 min. The filter paper was used to filter the supernatant to remove the lipids. DPA with various concentrations (0, 10, 20 and 50 μM) was spiked into the treated milk for further analysis, which then were added into the Eu^3+^/CDs sensing system. The fluorescence responses were measured under the excitation at 285 nm after incubating for 10 min.

### 2.5. The Smartphone-Integrated Assay for DPA Detection

For the smartphone-based assay for DPA detection, the samples were incubated with Eu^3+^/CDs and stirred for 10 min. Then, the fluorescent image was recorded and analyzed using the Color Picker APP on the smartphone to access its RGB intensity for analysis. It is worth noting that the samples were placed in a dark lamphouse to avoid environmental interference.

## 3. Results

### 3.1. The Principle of Ratiometric Fluorescence Strategy for DPA Determination

**Figure 1:**
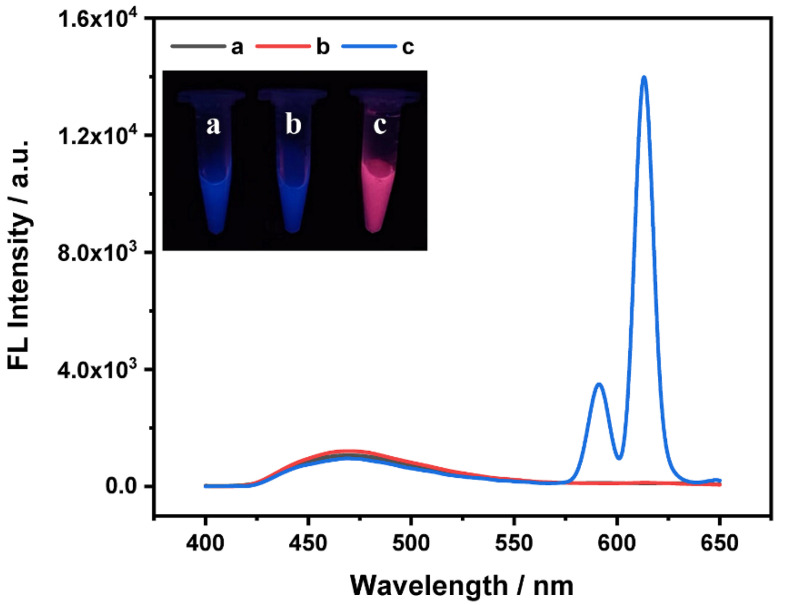
The fluorescence spectra and the corresponding photographs of CDs (a), CDs with Eu^3+^ (b), and Eu^3+^/CDs with DPA (c).

The ligand-containing DPA coordinated with Eu^3+^ and the triplet excited state of the ligand transferred energy to the emitting state of Eu^3+^ to enhance the fluorescence intensity (Figure S1 in Supplementary), which then directly detect DPA [30,31]. Considering that a single variation in signal was susceptible to environmental interference, a ratiometric fluorescent sensor was constructed to overcome these problems and improve the sensitivity and selectivity. As shown in Figure 1, the CDs emitted bright blue fluorescence at 470 nm (curve a), and its fluorescence intensity was not compromised when adding Eu^3+^ (curve b). The blue fluorescence can be obviously observed under the UV irradiation, seen in the insert photos. In the presence of DPA, the combined with the complex of Eu^3+^/CDs can obviously enhance the fluorescence at 615 nm due to the occurrence of antenna effect, while the blue fluorescence kept stability (curve c). The red fluorescence was easily found, seen in the insert photo. Therefore, the ratiometric strategy was established to sense the target based on the ratio of F_615_/F_470_ against increasing concentrations of DPA, which also provided the basis for intelligent detection using the smartphone with Color Picker APP. Here, the F_615_ and F_470_ were the fluorescence emission intensity of Eu^3+^/DPA at 615 nm and the CDs fluorescence emission intensity at 470 nm, respectively.

### 3.2. Optimization of Detection Experimental Conditions

**Figure 2:**
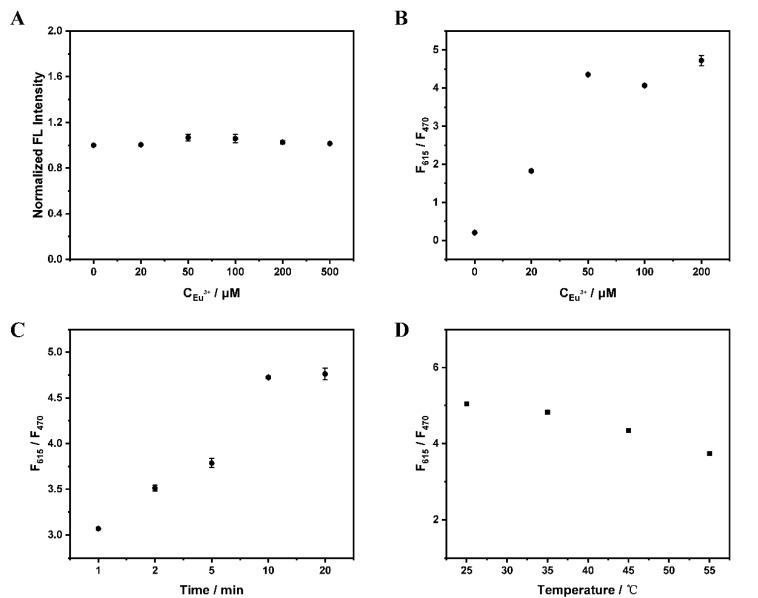
(**A**) The normalized fluorescence intensity of CDs against different concentrations of Eu^3+^. (**B**) The ratio of F_615_/F_470_ against different concentrations of Eu^3+^ in CDs with DPA. The reaction times (**C**) and different temperature (**D**) of the Eu^3+^/CDs system in the presence of DPA.

To guarantee the high sensitivity and stability of the Eu^3+^/CDs nanoplatform for target monitoring, several important factors were investigated and optimized, including the concentration of Eu^3+^, reaction temperature and incubation time. The concentration of Eu^3+^ was a key factor in the sensitivity of the assays. It can be seen that the blue fluorescent CDs maintains a stable fluorescence intensity with increasing the concentration of Eu^3+^ from 0 to 500 μM, which indicates that CDs can be used as an internal parameter (Figure 2A). Due to the energy transfer from DPA to Eu^3+^, the red fluorescence intensity increased significantly after adding different concentrations of Eu^3+^ (Figure 2B). The fluorescence ratio of F_615_/F_470_ was augmented with concentration of Eu^3+^ and reached a plateau at 50 μM. Here, the F_615_ and F_470_ were the emitted fluorescence intensity of Eu^3+^/DPA at 615 nm and the CDs at 470 nm, respectively. Thus, Eu^3+^ at 50 μM was selected for the detection of DPA in the following experiments. In order to investigate whether the experiment can be performed in the field for rapid detection, the temperature and reaction time of testing were crucial. Learn from Figure 2C,D, the reaction of Eu^3+^ and DPA can be carried out rapidly at room temperature and takes only 10 min, indicating that the system had the capability of detecting DPA in a short time and with high stability, providing an important guarantee for subsequent field testing.

### 3.3. The DPA Detection with Eu^3+^/CDs Probe

**Figure 3:**
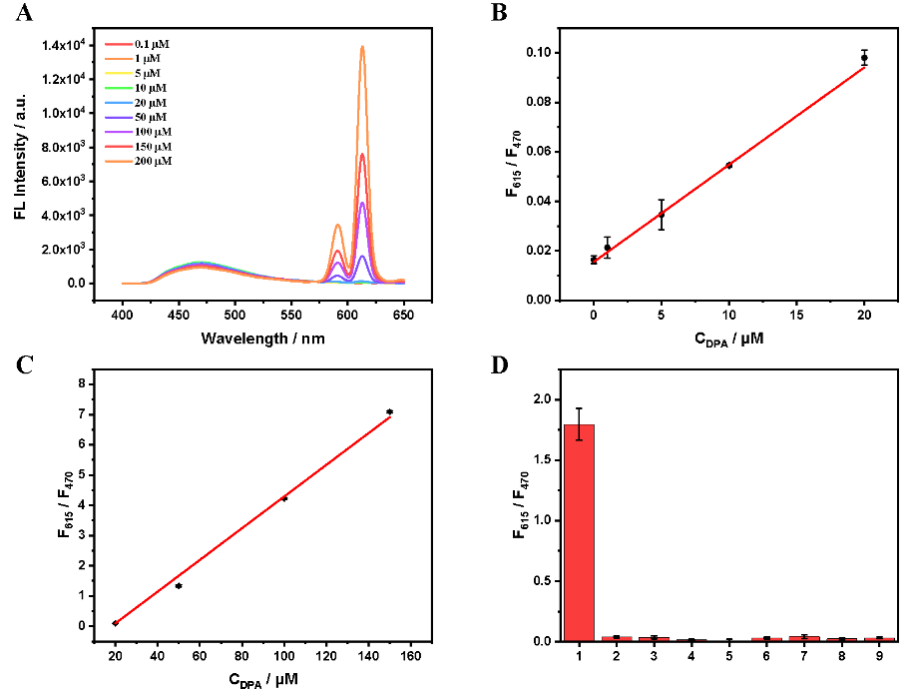
(**A**) The fluorescence spectra of Eu^3+^/CDs in the presence of different concentrations of DPA. The standard curves of DPA detection in the range from 0–20 μM (**B**) and 20–150 μM (**C**). (**D**) Selectivity sensing of the ratiometric fluorescence platform in the presence of each interferential substance and DPA target (1: DPA, 2: Cys, 3: GSH, 4: ATP, 5: Glu, 6: Cl^−^, 7: Fe^3+^, 8: Zn^2+^, 9: Ni^2+^).

Under the optimal reaction conditions, the detection sensitivity of the as-prepared Eu^3+^/CDs probe was examined by monitoring the ratio fluorescence response of the detection system as a function of different concentrations of DPA in Figure 3A. As the concentration of DPA increased from 0 to 200 μM, the coordination complex Eu^3+^/DPA was formed, resulting in augmented red fluorescence, while the blue emission fluorescence remained relatively constant. To evaluate the sensitivity of the ratiometric fluorescence platform, the ratio of F_615_/F_470_ was plotted against different concentrations of DPA, and the variation of ratio in Figure 3B,C was linear over the concentration range of 0–20 μM with a correlation coefficient R^2^ of 0.9961 and 20–150 μM with a correlation coefficient R^2^ of 0.9971. The limit of detection (LOD) was calculated to be as low as 1.18 μM according to the three times of signal-to-noise ratio, which confirmed the feasibility of the developed platform. The selectivity was further examined by measuring the fluorescence ratio of the system against other potential interfering analytes. As illustrated in Figure 3D, the fluorescence ratio of the target analyte DPA (50 μM) can be obviously monitored, which was significantly higher than that of other analytes, which proves that only DPA target can cause the change of sensor signal. Therefore, the developed strategy was specific and selective for the detection of DPA and can greatly reduce the influence of other interferents.

### 3.4. Sensitive Detection of DPA in Real Samples

To further explore the practicality of the proposed strategy, the milk samples with spiking a certain concentration of DPA were selected for the experiment. As displayed in Table 1, no DPA was detected in real samples. Then, the DPA with different concentrations (0, 10, 20, 50 μM) was spiked into the samples and then the same procedure was followed for quantification by the developed platform. The results showed satisfactory recoveries (97–99%) and accuracy (RSD < 4.05%) for detection of DPA, suggesting that the proposed platform can be used to determination the DPA in real food samples.

### 3.5. Smartphone-Integrated DPA Detection in Real Samples

**Figure 4:**
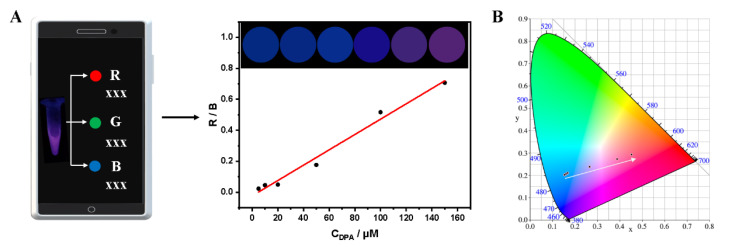
The smartphone-based sensor calibration plot based on ratio of R/B (**A**) and the CIE chromaticity coordinates of Eu^3+^/CDs (**B**) against concentrations of DPA range from 5 to 150 μM (Illustration: converted photos of Eu^3+^/CDs with different concentrations of DPA).

The current fluorescence assays usually require fluorescence spectrometer and specialized researchers, which is not suitable in on-site analysis [32,33,34,35,36,37]. In this research, a smartphone installed a Color Picker APP is used as a signal reader to detect the DPA by integrating with the as-prepared ratiometric fluorescent sensing, Figure 4A. The color of sample-triggered fluorescence photograph can be converted to digital values representing the blue (B), green (G) and red (R) color channels. The blue fluorescence (Blue channel) can gradually convert red fluorescence (Red channel) with increasing concentration of DPA range from 5 to 150 μM under UV light irradiation. Meanwhile, CIE coordinates can also prove the color changes from blue to red in Figure 4B. As shown in Figure 4A, the ration of red (G) to blue (B) channel (R/B) was linearly related in the range of 5 to 150 μM.

## 4. Conclusions

In summary, a ratiometric fluorescence strategy has been developed for sensitive and selective determination of DPA based on lanthanides-based Eu^3+^/CDs fluorescent sensor. In addition, the developed strategy can significantly reduce the interface from the complex matrixes and environments and that also improve the practicality and reliability. The determination of DPA is then qualified in real samples, showing the satisfactory recoveries from 97 to 99%. The obtained results of solution fluorescence within increasing concentration of DPA can be quantitatively analyzed by a smartphone loaded Color Picker APP, which can be applied to the POCT with accuracy and stability, exhibiting potential promising application in situ for DPA monitoring.

## Figures and Tables

**Table 1:** Determination of spiked samples of DPA in real samples by the developed strategy.

Sample	Spiked (μM)	Found	Recovery (%)	RSD (%)
Milk	0	-	-	-
	10	9.9	99	1.65
	20	19.8	99	4.05
	50	48.5	97	2.9

## Data Availability

Not applicable.

## Supplementary Materials

The following supporting information can be downloaded at: https://www.mdpi.com/article/10.3390/bios12080668/s1, Figure S1: The fluorescence spectra of (a) Eu^3+^, (b) DPA, and (c) Eu^3+^/DPA.
