# Supplementary material for: A Smartphone Integrated Platform for Ratiometric Fluorescent Sensitive and Selective Determination of Dipicolinic Acid
Source: Biosensors (Basel). 2022 Aug 22;12(8):668. doi: 10.3390/bios12080668 (PMC9405621; doi:10.3390/bios12080668)
Supplement: Supplementary file 1 [file biosensors-12-00668-s001.zip › biosensors-1826810-supplementary.pdf]

biosensors

Supplmentary

A Smartphone Integrated Platform for Ratiometric Fluorescent Sensitive and Selectivity Determination of DPA

Xiang Li [1,*], Junsong Wu [2], Huaguang Hu [1], Fangfang Liu [1] and Jialian Wang [1,*]

[1] School of Marine and Biological Engineering, Yancheng Teachers University (YCTU), Jiangsu, China.
[2] Department of Basic Medical Science, Jiangsu Vocational College of Medicine, Yancheng, Jiangsu, China, 224005, China.
* Correspondence: Lix01@yctu.edu.cn (X. L.); jlw0901@163.com (J. L.)

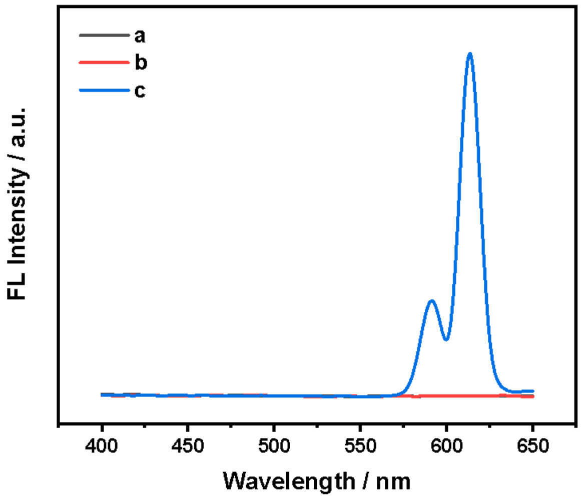

Figure S1. The fluorescence spectra of (a) Eu^{3+}, (b) DPA, and (c) Eu^{3+}/DPA.
